# The Role of TikTok in Education on Hidradenitis Suppurativa in Skin of Color: Cross-Sectional Analysis

**DOI:** 10.2196/71566

**Published:** 2026-02-27

**Authors:** Arsema K Zadu, Jordan Young, Janyla A Seltzer, Angel S Byrd, Cheri Frey

**Affiliations:** 1 Department of Dermatology, College of Medicine, Howard University, 2041 Georgia Ave NW, Washington, DC, 20060, United States, 1 202-865-6725

**Keywords:** TikTok, skin of color, hidradenitis suppurativa, patient education, social media

## Abstract

This study analyzed 50 TikTok videos returned by a search for “hidradenitis suppurativa in Black skin,” revealing that nearly half were patient-created, few had physician involvement (n=10, 20% dermatologists; n=7, 14% plastic surgeons), and few had commission-based (n=7, 14%) or sponsored content (n=2, 4%); they were predominantly patient testimonials on various treatments, highlighting the need for greater physician engagement to address patient needs, hidradenitis suppurativa product safety, and efficacy.

## Introduction

TikTok has become a prominent platform and search engine for dermatologic information [1]. A majority of videos on the top 20 most commonly diagnosed skin conditions were created by patients [2]. This study aimed to evaluate the primary sources of education on hidradenitis suppurativa (HS) in Black skin on TikTok.

HS is a chronic inflammatory condition marked by sinus tracts, nodules, and abscesses in intertriginous areas [3]. It often affects African American women and is frequently misdiagnosed, leading to delayed identification, accelerated progression, and treatment challenges [4]. Black patients experience a 1.5-year longer diagnostic delay than White patients, and after diagnosis, they typically wait 5 years to see a dermatologist compared to 3 years for White patients [5]. Early intervention is critical to prevent advanced-stage manifestations, which are often refractory to standard therapeutic approaches [6].

A study on dermatologist visibility on TikTok found that most dermatologic education videos are made by individuals without formal medical training [7]. While these platforms allow patients to connect and share experiences with skin conditions, the risk of spreading misinformation is significant, potentially worsening disease severity and reducing treatment effectiveness. This study aimed to describe who produces HS TikTok content featuring Black skin and to quantify the treatments, products, and themes presented.

## Methods

TikTok was selected because users often turn to the platform as a general search tool, including for health topics related to HS [1]. The app was used to search for “hidradenitis suppurativa in black skin” in the search bar. A total of 50 videos were viewed one by one by a single reviewer to assess the creator’s role, brands or products discussed, key themes, and whether the products were part of paid sponsorships or if the creator received commission from sales.

### Ethical Considerations

All data were deidentified prior to analysis. User IDs, screenshots, images, and quoted content contained no personally identifiable information. Data were analyzed and reported in anonymized form only.

## Results

Of the 50 analyzed videos, 24 (48%) were created by patients. Board-certified dermatologists produced 10 (20%) videos, while board-certified plastic surgeons produced 7 (14%) videos. One (2%) video was created by a nurse practitioner. Beauty service providers accounted for 2 (4%) videos. The “other” category, which included dietitians, social workers, product creators, and creators with unclear professional titles, accounted for 6 (12%) videos. Board certification status was verified using creator profile biographies and linked professional websites.

Seven videos featured products associated with sales commissions, and 2 videos involved paid sponsorships. Treatment content represented the largest category, comprising 35 (70%) videos (Table 1). Fourteen (28%) of the 50 videos focused on explaining HS. Of these, 9 (18%) were created by health care professionals, 4 (12%) by patients, and 1 by a product creator. The remaining videos focused on living with HS and dietary approaches to symptom management.

**Table 1.** Video themes stratified by creator type across 50 analyzed videos.

	Video type, n				
Creator type	Education	Medical treatment	Over-the-counter treatment	Lifestyle	Mental health
Patient	4	4	14	4	3
Board-certified dermatologist	7	3	5	0	0
Board-certified plastic surgeon	1	2	3	0	0
Nurse practitioner	1	0	0	0	0
Beauty service provider	0	1	1	0	0
Other	1	0	3	0	0

Products appeared across multiple categories, including face masks and exfoliants, body scrubs and cleansers, acne treatments, topical oils, salves, body butters, creams, antiseptics, spot treatments, systemic medications, and hair or scalp care, as shown in Table 2. PanOxyl and Hibiclens washes were the most frequently recommended products, appearing in 5 (10%) and 6 (12%) videos, respectively. Magic Healer body butter was mentioned in 4 (8%) videos, and Humira was mentioned in 3 (6%) videos. CeraVe acne foaming cream cleanser, a turmeric kojic acid soap, and Tend Skin Solution were all separately mentioned in 2 (4%) videos. All other products were mentioned in one video each.

**Table 2.** Categories of products mentioned across 50 analyzed hidradenitis suppurativa–related videos.

Category	Products
Face masks and exfoliants	Skinfix Glycolic Renewing Mask, Cocokind Turmeric Mask
Body scrubs and cleansers	Skinfix body scrub, Olay body wash, Naturium Vitamin C body wash, lemon turmeric and kojic acid soap, Dr. Bronner’s Soap, turmeric kojic acid soap, Dial bar soap
Acne cleansers and treatments	PanOxyl, PanOxyl acne foaming wash, Inkey List 5 % Benzoyl Peroxide Cleanser, CeraVe acne foaming cream cleanser, Zapzyt, Tend Skin Solution
Topical oils and salves	Relief Natural Company, GuruNanda Tea Tree, Zunda Turmeric, black seed oil, vitamin E, clove water, Magic Healer body butter, Palmer’s Body Oil
Body butters, creams, and moisturizers	HS Body Butter, Fenty Skin Cream, Healing Ocean Cream
Antiseptics and antimicrobials	Hibiclens, Magic Healer Product
Specialty treatments and patches	Mighty Patch
Medications	Humira
Hair and scalp care	Head & Shoulders

## Discussion

Patients produced the majority of lifestyle-based and experiential content, while physicians concentrated on treatment education and procedural intervention. Commercial messaging remained isolated to product owners and patient testimonials, with minimal clinical creator involvement. This distribution highlights a content gap between medically accurate education and the lived experience narrative dominating public HS discourse on TikTok. Research shows that only about 20% of skin of color videos are created by board-certified dermatologists [8]. These findings highlight an opportunity for dermatologists on TikTok to engage with patients and address the safety and efficacy of these products. Dermatologists, who already have a strong social media presence, can foster patient trust by encouraging open discussions about non–medical-grade treatments. By acknowledging the value of herbal and alternative remedies that patients find helpful, they can assess their safety and efficacy while supporting their continued use when appropriate. Meanwhile, they can provide evidence-based guidance to minimize the risks of harmful or ineffective treatments.

## Abbreviations

HS: hidradenitis suppurativa
